# Intensity-modulated radiation therapy: emerging cancer treatment technology

**DOI:** 10.1038/sj.bjc.6602577

**Published:** 2005-04-26

**Authors:** T S Hong, M A Ritter, W A Tomé, P M Harari

**Affiliations:** 1 Department of Human Oncology, University of Wisconsin Medical School, Madison, WI, USA; 2 Department of Medical Physics, University of Wisconsin Medical School, Madison, WI, USA

**Keywords:** intensity-modulated radiation therapy, head and neck cancer, prostate cancer

## Abstract

The use of intensity-modulated radiation therapy (IMRT) is rapidly advancing in the field of radiation oncology. Intensity-modulated radiation therapy allows for improved dose conformality, thereby affording the potential to decrease the spectrum of normal tissue toxicities associated with IMRT. Preliminary results with IMRT are quite promising; however, the clinical data is relatively immature and overall patient numbers remain small. High-quality IMRT requires intensive physics support and detailed knowledge of three-dimensional anatomy and patterns of tumour spread. This review focuses on basic principles, and highlights the clinical implementation of IMRT in head and neck and prostate cancer.

Intensity-modulated radiation therapy (IMRT) represents a promising new advance in the field of radiation oncology. Intensity-modulated radiation therapy allows for improved ‘shaping’ of radiation dose profiles around tumour and at-risk nodal structures while sparing adjacent normal tissue structures. This capacity for improved dose distribution affords considerable opportunity to reduce the overall toxicity profile associated with radiation therapy. The use of IMRT is advancing rapidly worldwide as this technology has become commercially available. Despite considerable promise, IMRT use remains in relatively early stages, and must be delivered with strict attention to quality assurance as the clinical data and patient follow-up mature. Furthermore, IMRT is quite labour-intensive, with strong dependence on physics and quality assurance support, thus leaving open the possibility for significant heterogeneity in the precision of IMRT practice.

## 

### What is IMRT?

Intensity-modulated radiation therapy refers to a specific technique of linear accelerator-based radiation therapy whereby beams are modulated in such a manner to produce highly conformal dose distributions. A primary objective of IMRT is to reduce dose to selected normal tissue structures in an effort to preserve function, while maintaining full dose delivery to tumour targets. For example, in conventional head and neck (H&N) radiotherapy, static fields are shaped by blocks and modulated by simple beam wedges or tissue compensators (Harari *et al*, 1998). In contrast, IMRT is delivered by individually modulated fields (step and shoot or sliding window technique) or by a rotating linear accelerator gantry (serial or helical tomotherapy). Variable dose intensities can be delivered through the segments of each treatment field, thereby maximizing conformality of the ultimate dose distribution.

Intensity-modulated radiation therapy planning is conceptually distinct from conventional radiotherapy planning. For both conventional (3D conformal radiation therapy, 3D-CRT) and IMRT planning, the radiation oncologist designates specific targets (gross tumour, elective nodal regions) and avoidance structures (rectal wall, bladder, spinal cord, salivary glands, optic apparatus, etc.). In 3D-CRT, simple beam arrangements are used with generous field margins to account for daily set-up variation and physical characteristics of the beam itself. The radiation dose and profile is then calculated in a process known as *forward planning*. In contrast, IMRT planning requires that the physician define dose specifications for both the target and avoidance structures. The computer planning software then creates a series of modulation patterns at each beam angle that strive to achieve the physician's dose prescription goals. This process is known as *inverse planning*.

### Brief history of IMRT

Intensity-modulated radiation therapy was first conceptualised in the 1960s. However, it was not until the 1980s–1990s that the computing capability required for complex inverse planning algorithms became commercially available (Intensity Modulated Radiation Therapy Collaborative Working Group, 2001). In 1994, the NOMOS Peacock system was introduced as the first commercial IMRT delivery unit. The Peacock system required the use of a beam modulation device known as a dynamic multivane intensity-modulating collimator (MIMiC). This particular form of IMRT is called serial tomotherapy, as ‘slices’ could be treated by a continually rotating gantry (Intensity Modulated Radiation Therapy Collaborative Working Group, 2001). Step and shoot IMRT represents another commonly used technique whereby multiple static beams are subdivided into ‘segments’. In the sliding window technique, a window defined by the MLC leaves sweeps across the treatment field at variable speed, while the monitor units are delivered continuously (Ling *et al*, 1996). With serial and helical tomotherapy, the intensity modulation is achieved through the use of a binary MLC (radiation is either delivered or not). In contrast to serial tomotherapy, helical tomotherapy is characterised by translation of the treatment couch during treatment delivery, allowing large field lengths to be treated in a single spiral (Mackie *et al*, 1999). Moreover, a CT detector array diametrically opposed to the energy source allows for image and dose reconstruction capabilities during treatment. Each of these systems shares commonality of need for intensive physics support, precise anatomical target definition, and rigorous quality assurance (Richardson *et al*, 2003; Fenwick *et al*, 2004).

## CLINICAL IMPLICATIONS

Clinical research efforts in IMRT have generally considered two basic paradigms. The first research strategy seeks to maintain current tumour control rates while decreasing toxicity profiles. For example, IMRT studies in H&N cancer commonly strive to maintain conventional dose to primary tumour and at-risk nodal regions, while diminishing dose to adjacent normal tissue structures such as salivary glands and spinal cord. The second strategy attempts to escalate tumour target dose while maintaining acceptable levels of toxicity. This approach has been taken in dose-escalation and hypofractionation trials for prostate cancer and more recently in lung cancer. Regardless of the primary objective, precise, reproducible accurate patient/target set-up and rigorous physics quality assurance are critical to successful IMRT delivery. In this review, we highlight the role of IMRT in two of the most common anatomic sites of current use; H&N and prostate cancer.

### Clinical applications in H&N cancer

Radiation plays a central role in the treatment of H&N cancer. New radiation delivery techniques offer powerful potential to diminish the spectrum and severity of radiation toxicities for H&N cancer patients. For many decades, conventional H&N radiation techniques have involved treatment with generous opposed lateral beams to encompass the known primary tumour and upper cervical lymphatics. This classical technique produces a relatively homogeneous dose distribution that allows excellent target dosing while minimizing hot and cold spots. However, due to the tight proximity of tumour targets and normal tissue in the H&N region, many uninvolved structures including salivary glands, spinal cord, auditory apparatus, optic apparatus, mandible, and vocal cords can unnecessarily receive high doses of radiation.

In H&N cancer, one of the most common rationales for IMRT is to preserve salivary gland function and thereby diminish the severity of chronic xerostomia with associated adverse impact on taste, swallowing, dentition, speech, and overall quality of life. In addition, the capacity of IMRT to limit dose to normal tissue structures may also allow dose escalation and differential dose painting, thereby accomplishing ‘in-field tumour boosting’ (Butler *et al*, 1999).

Early reports have described clinical promise with H&N IMRT, both for tumour control and reduction of xerostomia. However, these data generally represent limited single-institution experience, often with heterogeneous cohorts of patients (postoperative *vs* definitive, chemotherapy *vs* no chemotherapy, varying dose/fractionation schemes). Prospective, multiinstitutional protocols that incorporate IMRT for H&N cancer patients remain in very early stages.

More mature clinical data regarding the efficacy of IMRT in the management of H&N cancer is now emerging. Preliminary single-institution experience with IMRT suggests favourable outcome. While these results can be interpreted with caution, given the small overall numbers and careful patient selection, they suggest that H&N IMRT appears to be safe and effective in appropriately selected patients.

The Washington University experience with H&N IMRT included 126 patients (Chao *et al*, 2003). In total, 41% of patients were treated definitively and the remainder postoperatively. The 2-year actuarial locoregional control rate was 85%. Patterns of recurrence from patients treated at the University of Michigan with parotid sparing H&N IMRT techniques were recently reported (Dawson *et al*, 2000). A total of 58 patients with primary H&N cancer were treated definitively or postoperatively and followed for a median of 27 months. A 79% local rate of control was achieved with 12 patients developing recurrence by 2 years. Investigators at Baylor reported on 20 patients with primary H&N tumours showing 19 patients with a complete response to therapy and a significant reduction of dose to parotid glands (Butler *et al*, 1999). With a median follow-up of 13.5 months, two patients who achieved complete response developed local recurrence at 10 and 15 months. Treatments were generally well tolerated. Lee *et al* (2002) reported on 67 patients with nasopharyngeal carcinoma who were treated with IMRT. Of 58 patients, 50 were treated with concomitant and adjuvant chemotherapy. With a median follow-up of 31 months, a local regional progression-free rate of 98% was observed. A recent update with 118 patients continues to demonstrate excellent locoregional control rates (Bucci *et al*, 2004). These results have stimulated further evaluation of H&N IMRT in an ongoing cooperative group trial for nasopharynx cancer patients through the Radiation Therapy Oncology Group.

**Figure 1:**
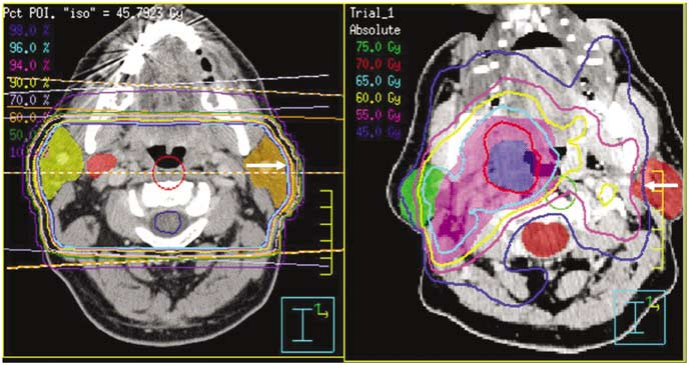
Isodose distributions contrasting conventional (left) and IMRT (right) H&N treatment plans. Significant reduction of dose to the left parotid gland is achieved with the IMRT plan.

Xerostomia brings significant long-term consequences for the H&N cancer patient. Lack of salivary production can lead to sore throat, decreased taste, dental decay, mandibular osteoradionecrosis, and impaired voice and swallowing functioning. Intensity-modulated radiation therapy techniques afford distinct opportunities for salivary gland sparing (Figure 1). Investigators at the University of Michigan suggest that mean doses of ⩽26 Gy to the parotid gland may afford substantial sparing of long-term parotid gland function. At ⩽26 Gy, excellent preservation of salivary function (unstimulated and stimulated, respectively) was observed (Eisbruch *et al*, 1999). This group has also demonstrated that salivary flow correlates with improved quality of life, suggesting that parotid sparing may be associated with improved overall clinical outcome (Eisbruch *et al*, 2001). Chao *et al* (2001) have reported on results of a trial examining the functional outcome of salivary glands at 6 months following radiation. Mean dose to the parotid gland was shown to correlate with ultimate salivary flow in 41 patients analysed. More recent studies continue to suggest that salivary sparing is possible with IMRT using proper technique (Astreinidou *et al*, 2004) and may favourably impact overall quality of life (Reddy *et al*, 2001; Parliament *et al*, 2004).

While these studies suggest that IMRT represents a promising new therapy in H&N cancer, early results must be viewed with some caution. Aside from the nasopharynx data, these series include a heterogeneous group of H&N patients, some treated definitively and some postoperatively. Chemotherapy regimens and fractionation regimens vary within and across the published series. Overall follow-up for the patients remains relatively short. The specific techniques of IMRT treatment show evolution within each of these updated series. These factors suggest that ongoing careful and systematic evaluation regarding acute and long-term outcomes with H&N IMRT should be pursued. Data are emerging regarding key contributing factors for disease control and treatment failure for H&N IMRT, including tumour characteristics and treatment technique. Chao *et al* (2004) identified that primary tumour GTV and nodal GTV size independently predicted for therapeutic outcome. Patterns of failure analysis in patients treated with IMRT led Eisbruch *et al* (2004) to recommend careful attention to retropharyngeal nodes in patients with oropharyngeal primaries. Further reports based on clinical outcome will continue to shape the practice of H&N IMRT.

### Clinical applications in prostate cancer

The past decade has provided new data regarding of the importance of dose escalation in the treatment of localised prostate cancer. Pollack *et al* (2002) reported results of a seminal phase III study of dose escalation at the MD Anderson Cancer Center. Patients with low- or intermediate risk localised prostate cancer were randomised to 70 *vs* 78 Gy. The high dose arm showed a statistically significant improvement in freedom-from-failure. These results are further supported by several single-institution series (Zelefsky *et al*, 1998a, 1998b; Pollack *et al*, 2004).

**Figure 2:**
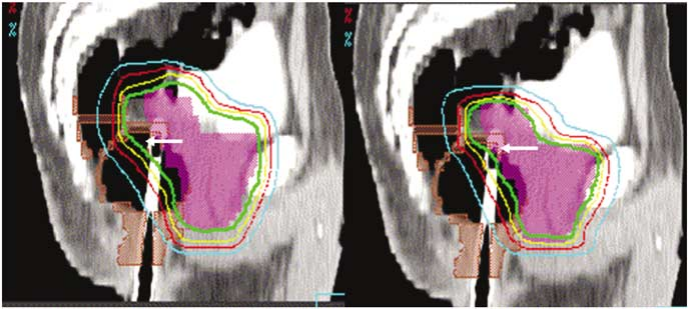
Isodose distributions contrasting conventional 3D conformal (left) and IMRT (right) prostate treatment plans. The arrows highlight improved conformality to the prostate and seminal vesicles and decreased rectal volume receiving high dose with the IMRT plan.

Escalation of radiation dose to the prostate brings increased toxicity risks, particularly rectal complications. When delivered with conventionally planned techniques, doses higher than 70 Gy are associated with higher complication rates (Pollack *et al*, 2002; Tucker *et al*, 2004). It has now become clear that 3D conformal radiotherapy techniques allow improved overall treatment tolerance of higher doses (Nguyen *et al*, 1998; Zelefsky *et al*, 1998a, 1998b; Michalski *et al*, 2000) but complication rates, particularly rectal bleeding, can remain substantial. Several analyses suggest that the total volume of rectal wall exposed to greater than 60–70 Gy predicts the rate of grade 2 (rectal bleeding) or more severe complications. Therefore, the implementation of IMRT, with the ability to further reduce rectal dose (Figure 2), should further reduce toxicities, as has recently been reported (Zelefsky *et al*, 2002).

Increasing the conformality of radiation dose requires increased set-up precision. In recent years, transabdominal ultrasound systems have been employed to more accurately target the prostate on a daily basis (Lattanzi *et al*, 1999; Lattanzi *et al*, 2000; D'Amico *et al*, 2001). As an alternative, the implantation of small metal seeds into the prostate to serve as fiduciaries during daily portal imaging has also proven reliable as a means of reproducibly localising the prostate (Nederveen *et al*, 2003). In addition, it has been demonstrated that the use of a rectal balloon catheter can immobilise the prostate and facilitate localisation on port films potentially allowing tighter margins for the treatment volume (Wachter *et al*, 2002). Further, the lateral and posterior aspects of the rectal wall are partially displaced out of the high dose region by the rectal balloon, which offers the potential for significant rectal dose sparing (Patel *et al*, 2003).

A number of centres have sought to reduce the number of fractions required for prostate cancer treatment by increasing fraction size. While the rationale has been primarily for logistical and resource utilisation purposes in the past, it has recently become better appreciated that prostate cancer may have unique radiobiological properties (increased ability to repair damage). It may, therefore, prove advantageous to use larger daily fractions of >2.0 Gy (hypofractionation), rather than conventional 1.8–2.0 Gy fractions commonly employed, when treating most other tumour types. Using linear quadratic formulation, one can predict an improved ratio of tumour control to normal tissue toxicity (the therapeutic ratio) for prostate cancer by using hypofractionation (Fowler *et al*, 2001). Cleveland Clinic investigators have carried out a trial of hypofractionation consisting of 28 fractions of 2.5 Gy for a total dose of 70 Gy (Kupelian *et al*, 2002). Intensity modulated radiotherapy and daily pretreatment ultrasound-based prostate localisation were employed to improve treatment precision and reduce radiation dose to the rectum. These early results appear to indicate disease control (PSA recurrence-free survival) at least equivalent to that seen with standard fractionation, although follow-up remains short. Given the smaller number of larger radiation doses delivered, highly conformal and accurate radiation techniques like image-guided IMRT afford the opportunity for the exploration of novel fractionation regimens, which ultimately may improve local control and cost-effectiveness. However, with the delivery of fewer treatment fractions, errors incurred through daily set-up variations or internal organ motion with each fraction will have a larger impact on overall treatment, and therefore there is clear need for high precision patient localisation techniques with such treatment strategies (Keller *et al*, 2004; Orton and Tome, 2004).

### IMRT precautions

As with any new technology, there is enthusiasm mixed with caution regarding the use of IMRT. Some have voiced concern regarding the embracement of IMRT as a standard approach until the completion and comparative evaluation of systematic clinical trials (Halperin, 2000; Glatstein, 2003). Nevertheless, the global use of IMRT has increased dramatically over the last several years. In a recent survey of US radiation oncologists published by Mell *et al* (2003), one-third of respondents reported that they were currently using IMRT. In addition, over 90% of respondents who were not currently using IMRT stated that they planned to do so in the near future. Despite increased utilisation, several notes of caution regarding the use of IMRT are worthy of mention.

#### IMRT standardisation

**Figure 3:**
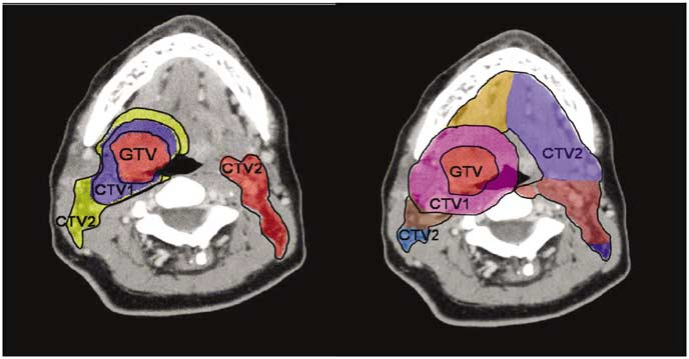
Variations in H&N target delineation. Highly distinct CTV designs from two H&N experts which illustrate broad variation in target delineation strategies for the identical tonsil case.

There exists a lack of global standardisation in IMRT planning and delivery. The literature describes several distinct IMRT techniques, and several aspects of the IMRT planning processes remain highly practitioner dependent. A variety of fractionation regimens and target delineation techniques are in common use. In a recent study, substantial variation in both target delineation and fractionation recommendations were observed among worldwide H&N experts when provided the identical tonsil cancer case (Figure 3) (Hong *et al*, 2004). Subtle technique distinctions can pose considerable challenge to new institutions that wish to commence use of IMRT. For these reasons, it is increasingly important to provide standardised recommendations and guidelines for IMRT planning. Indeed, guidelines are beginning to emerge with specific recommendations for nodal coverage targets based on tumour location and stage (Eisbruch *et al*, 2002; Gregoire *et al*, 2003; Levendag *et al*, 2004).

#### IMRT set-up precision

Radiation oncologists have been traditionally trained to use large field margins to cover unsuspected tumour infiltration and to avoid geographical miss. Since a major goal of IMRT is to limit dose to normal tissue structures that often reside very close to tumour targets, daily set-up precision takes on much greater significance. A recent study suggests that the daily set-up variations with conventional H&N masking and immobilisation techniques may be insufficient to ensure high-quality IMRT delivery over a 6–7 week course of treatment (Hong *et al*, 2005). Indeed, daily set-up errors of several mm can result in underdosing of tumour or overdosing of normal tissues such as the ‘spared’ parotid gland, underscoring the importance of rigorous quality assurance processes for IMRT.

#### Radiation exposure

Another theoretical concern with IMRT involves the increased machine output (monitor units) required for IMRT delivery and the potential for increased radiation exposure. With increased modulation of the radiation beam, more monitor units are generated to deliver the prescribed dose. Consequently, there can be increased leakage from the linear accelerator, increasing the total body exposure by 2–3 fold (Hall and Wuu, 2003). In a recent publication, Hall *et al* suggest that this increase in total body dose could potentially increase the rate of second malignancy from 1% per 10 years to 1.75% per 10 years, an almost doubling of the second malignancy rate. Careful follow-up will be required to determine if IMRT does in fact enhance this risk.

#### Future IMRT trials

As H&N IMRT steadily advances into more common use, the design of clinical trials that explore the use of chemoradiation, altered fractionation, and molecular targeted therapy becomes more complex. Indeed, the cooperative oncology groups are struggling currently with systematic methodology to credential and quality-assure the process of H&N IMRT for participating institutions. The successful accomplishment of future H&N cancer treatment trials will need to acknowledge the increasing use of IMRT despite inherent difficulties in trial design and quality assurance. This represents a significant challenge in that a broad series of promising molecular agents, that may enhance radiation response are becoming available. However, the profound variation in IMRT expertise and delivery technique across institutions currently renders this an added variable that serves to complicate the evaluation of new molecular agents with radiation.

In prostate cancer, novel hypofractionated regimens have been proposed to exploit radiobiological properties with significant shortening of treatment time without loss of efficacy or increase in toxicity (Fowler *et al*, 2003). Indeed, investigators are exploring the utility of IMRT in a number of other clinical scenarios, including brain tumours, lung cancer, upper GI malignancies, breast cancer, gynaecologic malignancies, and many other sites. The primary objectives focus on reduction of toxicity and/or increased dose to the tumour target.

Other new aspects of IMRT research include the integration of sophisticated image-guidance. As discussed previously, the steep dose gradients created by IMRT plans can increase the risk of geographical target miss or unintentional overdosing of spared structures. Recent technologies such as ultrasound position verification, cone beam CT, and MVCT with helical tomotherapy offer methods to ‘visualise’ daily treatment set-up and reduce set-up variability and resultant dosimetric uncertainty (Mackie *et al*, 2003). Indeed, high-quality IMRT is highly dependent on accurate and reproducible patient set-up.

### Future directions and conclusions

There is no question that the use of IMRT in modern cancer therapy is expanding worldwide. This promising technology advancement brings clear opportunity to improve the therapeutic ratio for cancer patient outcome. The early clinical reports to date are quite promising for several distinct tumour types. However, improved definition of those specific parameters (tumour and normal tissue) that render patients most likely to truly benefit from IMRT will be valuable. The time, expense, and expertise required to realise optimised and reproducible IMRT delivery across institutions warrants acknowledgement regarding the most appropriate usage and necessary quality assurance processes to ensure patient benefit and safety. The systematic accumulation of clinical data is of great importance for advancement in this field. Ideally, this data will come in the form of controlled clinical trials that rigorously examine not just radiation physics and dosimetry, but acute and late normal tissue effects with long term clinical follow up.
